# Innate immunity limits protective adaptive immune responses against pre-erythrocytic malaria parasites

**DOI:** 10.1038/s41467-019-11819-0

**Published:** 2019-09-02

**Authors:** Nana K. Minkah, Brandon K. Wilder, Amina A. Sheikh, Thomas Martinson, Lisa Wegmair, Ashley M. Vaughan, Stefan H. I. Kappe

**Affiliations:** 1 Center for Global Infectious Disease Research, Seattle Children’s Research Institute, 307 Westlake Ave N, Seattle, WA USA; 2 Department of Global Health, University of Washington, Seattle, WA USA; 3 Present Address: Vaccine and Gene Therapy Institute, Oregon Health and Science University, Portland, OR USA; 4 Present Address: Santis GmbH/Astrazeneca, Leverkusenstasse 54, 22761 Hamburg, Germany

**Keywords:** Immunological memory, Interferons, Malaria, Live attenuated vaccines

## Abstract

Immunization with attenuated whole *Plasmodium* sporozoites constitutes a promising vaccination strategy. Compared to replication-deficient parasites, immunization with replication-competent parasites confers better protection and also induces a type I IFN (IFN-1) response, but whether this IFN-1 response has beneficial or adverse effects on vaccine-induced adaptive immunity is not known. Here, we show that IFN-1 signaling-deficient mice immunized with replication-competent sporozoites exhibit superior protection against infection. This correlates with superior CD8 T cell memory including reduced expression of the exhaustion markers PD-1 and LAG-3 on these cells and increased numbers of memory CD8 T cells in the liver. Moreover, the adoptive transfer of memory CD8 T cells from the livers of previously immunized IFN-1 signaling-deficient mice confers greater protection against liver stage parasites. However, the detrimental role of IFN-1 signaling is not CD8 T cell intrinsic. Together, our data demonstrate that liver stage-engendered IFN-1 signaling impairs hepatic CD8 T cell memory via a CD8 T cell-extrinsic mechanism.

## Abstract (web summary)

Here, Minkah *et al*. show that, while immunization with replication-competent *Plasmodium* parasites can confer sterile protection against infection, it also induces a type I interferon response that adversely affects anti-malaria immunity by affecting numbers of protective hepatic CD8 T cells and CD8 T cell function.

## Introduction

Infection by *Plasmodium* parasites causes more than 200 million malaria clinical cases and results in over 400,000 deaths annually, mainly in pregnant women and children under the age of five. No fully protective vaccine exists. *Plasmodium* parasite genomes encode over 5000 genes which are differentially transcribed as the parasite progresses through its vector and mammalian host multi-organ life cycle, rendering *Plasmodium* a difficult target for traditional subunit vaccine approaches that have been successful for less complex pathogens. The mammalian stages of infection are initiated when sporozoites are injected into the skin by female *Anopheles* mosquitoes^1^. Sporozoites traverse multiple cell types in the skin, access capillaries, and transit to the liver. Here, each sporozoite infects a single hepatocyte, transforms within and develops as a liver stage (LS), undergoing multiple rounds of genome replication, to produce tens of thousands of red blood cell-infective exo-erythrocytic merozoites.

Merozoites are released into the blood where they infect red blood cells, replicate within, and are released, thereby undergoing continuous cycles of infection, replication, and release, allowing parasite numbers in the blood to reach billions. The sporozoite and LS of infection (referred to as the pre-erythrocytic stages) are asymptomatic while all malaria-associated morbidity and mortality is associated with the blood stages of infection^2^. Immunization with whole attenuated sporozoites unable to cause blood stage infection constitute an attractive vaccine strategy. These strategies include radiation-attenuated sporozoites (RAS), the administration of sporozoites under anti-blood stage drug cover (known as infection treatment vaccination or ITV) and genetically attenuated parasites (GAP) in which parasite arrest is mediated by the targeted deletion of parasite genes critical for LS development^3,4^. GAPs have the advantage that targeted gene deletion can determine the degree of parasite replication competence^5^. Moreover, attenuation by genetic engineering allows for further modification of the whole sporozoite immunogen to enhance immunogenicity and subsequent vaccine efficacy. GAPs confer sterile protection in rodents and data from a recently published Phase I clinical trial testing the safety profile of a first-generation early LS-arresting (EA) replication-deficient (RD) *P. falciparum* GAP showed that GAPs are safe and can engender potent immune responses to sporozoite antigens^6^. Furthermore, in animal models, late LS-arresting (LA), replication-competent (RC) *Plasmodium yoelii* GAPs afford superior pre-erythrocytic immunity as well as stage- and strain- transcending immunity^7–9^ as compared to EARD GAPs and RAS. In humans, the superior immunogenicity of RC whole sporozoite vaccines is demonstrated by the observation that in comparison to RAS, ITV requires a fraction of an immunizing sporozoite dose to achieve complete sterilizing protection against controlled human malaria infection^10^.

In mouse models of *Plasmodium* infection, adaptive immune responses engendered by whole sporozoite immunization have been extensively studied. Antibody responses significantly contribute to protection^8,11–15^. Unlike antibodies, however, CD8 T cells alone are capable of conferring complete sterilizing protection, indicating their critical role in pre-erythrocytic immunity^16–19^. Recently, we and others reported that live parasite infection and replication in hepatocytes induces an innate immune response that is dependent on type I IFN (IFN-1) signaling^20,21^. However, it remains unknown whether this IFN-1 response is beneficial, detrimental, or has no effect on vaccine-induced adaptive immunity. Given the well-established favorable roles of IFN-1 signaling on the development of adaptive immunity^22–24^, we hypothesized that the enhanced adaptive protection afforded by LARC GAP immunization was in part dependent on their ability to elicit this potent innate immune response. However, we here report the observation that the parasite-engendered IFN-1 response in fact dampens adaptive CD8 T cell immunity and vaccine-engendered protection. This impaired protection correlates with a reduction in the magnitude and quality of memory CD8 T cells in the liver after immunization of mice, which we investigated further.

## Results

### Immunized IRF3^−/−^ and IFNAR^−/−^ mice exhibit superior protection

Rodent malaria LARC GAP have been generated by deletion of genes encoding enzymes in the endogenous type II fatty acid biosynthesis pathway, including FabB/F. These parasites suffer a growth defect late during LS schizogony, do not form exo-erythrocytic merozoites, and cannot initiate blood stage infection^25^. Rodent infection with LARC GAP or wild-type (WT) parasites induces an innate immune response initiated by the IRF3 transcription factor and propagated through the IFN-1 receptor, IFNAR^20,21,26^.

**Fig. 1:**
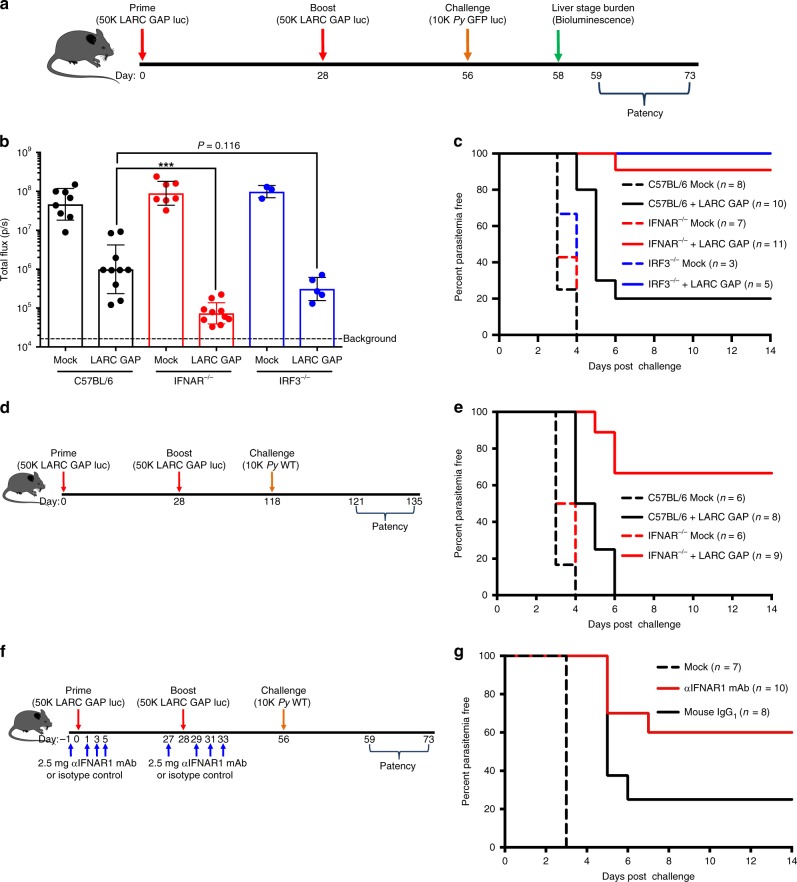
Ablation of type I IFN signaling enhances protection against pre-erythrocytic *Plasmodium* infection in LARC GAP-immunized mice. **a** Schematic of the immunization regimen. Mice were immunized twice with 50,000 LARC GAP sporozoites and then challenged with 10,000 *Py* GFPluc sporozoites 1 month after the final immunization. **b** Quantification of bioluminescent LS parasite burden 44 h after challenge. LS burden is significantly reduced in the livers of immunized WT B6 mice and further reduced in IFNAR^−/−^ mice and IRF3^−/−^ mice. **c** Examination of blood stage infection in immunized mice after sporozoite challenge 30 days post last immunization. **d** Schematic of the immunization regimen with first challenge at 90 days after the last immunization. Mice were immunized with LARC GAP sporozoites using the immunization regimen outlined in **a** and challenged with *Py* WT sporozoites 3 months after the last immunization. **e** Examination of blood stage infection in immunized mice after sporozoite challenge 90 days after thelast immunization. **f** Schematic of GAP-immunization regimen after treatment with αIFNAR1-blocking mAbs. **g** Examination of blood stage infection in B6 immunized mice treated with an IFNAR1-blocking mAb or isotype control antibody and challenged with WT sporozoites 30 days after the last immunization. Data from panels **b**, **c**, **e** and **g** are compiled from two independent experiments with at least two mice in each group per experiment. Each dot represents a single mouse. Bar graphs are expressed as mean ± SD. Total number of mice in each experiment is shown in the survival curves. ***p < 0.0001 (from unpaired two-tailed Student’s *t*-test). Components of this figure were created using Servier Medical Art templates, which are licensed under a Creative Commons Attribution 3.0 Unported License; https://smart.servier.com

To test the impact of IFN-1 signaling on adaptive immunity, we immunized C57BL/6 WT (B6), IRF3^−/−^, and IFNAR^−/−^ mice with a partially protective regimen of two doses of 50,000 *P. yoelii (Py) fabb/f*^–^ LARC GAP sporozoites. Four weeks after the second immunization, mice were intravenously (i.v.) challenged with 10,000 *Py* GFPluc (luciferase-expressing WT *P. yoelii* 17XNL)^27^ (Fig. 1a). Forty-four hours after challenge, we quantified parasite LS burden by bioluminescent imaging. Compared to mock-immunized mice, B6 mice immunized with LARC GAP showed a 34-fold reduction in parasite LS burden (Fig. 1b). Immunized IFNAR^−/−^ and IRF3^−/−^ mice showed greater reductions of LS burden of 1995- and 162-fold, respectively, although the latter approached but did not reach statistical significance (Fig. 1b). This enhanced protection against LS infection in immunized mice lacking competent IFN-1 signaling was reflected in the lack of onset of blood stage infection (patency) (Fig. 1c). While only 20% of the immunized B6 mice were completely protected, with the remaining 80% exhibiting an approximate 2-day delay in the onset of blood stage patency (Fig. 1c), 90% of the immunized IFNAR^−/−^ mice and 100% of immunized IRF3^−/−^ mice were completely protected as they exhibited no signs of blood stage infection (Fig. 1c). To examine the durability of LARC GAP-engendered protection in the context of IFN-1 signaling, we immunized cohorts of B6 and IFNAR^−/−^ mice and quantified patency upon *Py* GFPluc challenge 3 months after the last immunization (Fig. 1d). None of the B6 mice immunized with LARC GAP were completely protected although they exhibited a 1- to 2-day delay in the onset of patency, indicating a partial reduction of LS burden (Fig. 1e). In contrast, 75% of the immunized IFNAR^−/−^ mice were completely protected.

To confirm these results were due to the lack of IFNAR signaling, we evaluated protection in LARC GAP-immunized B6 mice that received treatment with an IFNAR1-blocking monoclonal antibody (mAb) or its isotype control at the time of immunization using the same immunization-challenge regimen as before (Fig. 1f). In agreement with the results in IFNAR^−/−^ mice, i.v. challenge of each group with 10,000 *Py* WT sporozoites 4 weeks after the last immunization resulted in sterile protection in 60% of the LARC GAP immunized mice that received the IFNAR1 mAb while only 20% of the isotype control-treated mice were protected (Fig. 1g).

Next, we set out to determine whether the negative impact of the innate IFN-1 response on protective adaptive immunity was also observed using a different RC vaccination type, ITV. For this, we examined protection in B6 mice and IFNAR^−/−^ mice immunized with *Py* GFPluc sporozoites and subsequently treated twice with atovaquone at 30- and 40-h post infection (hpi) to prevent the completion of LS development and subsequent blood stage infection (Supplementary Fig. 1A). All immunized mice were challenged with *Py* WT sporozoites 4 weeks after the last immunization. None of the immunized B6 mice were completely protected but they exhibited a delay in the onset of blood stage patency. In contrast, 40% of the immunized IFNAR^−/−^ mice were completely protected and the remaining 60% exhibited a 3-day delay to the onset of blood stage patency (Supplementary Fig. 1B). Our data indicate that the efficacy of the immune response engendered after immunization with two distinct whole sporozoite vaccinations, LARC GAP and ITV, is enhanced in the absence of IFN-1 signaling.

We also examined whether IFN-1 signaling impacted the development of adaptive immunity after immunization with RD whole sporozoite vaccines. We immunized B6 mice with the EARD GAP (*p52*^−^/*p36*^−^/*sap1*^−^)^6^ and treated each mouse with either an IFNAR1-blocking monoclonal antibody or its isotype control at the time of immunization (Supplementary Fig. 1C). All mice were challenged with *Py* GFPluc sporozoites 28 days after the last immunization. We did not observe a difference in parasite LS burden (Supplementary Fig. 1D) or time to patency (Supplementary Fig. 1E) between groups. Furthermore, no difference in time to patency was observed after EARD GAP immunization of WT or IRF3^−/−^ mice (Supplementary Fig. 1F, 1G).

Lastly, we examined whether the enhanced protection observed in LARC GAP-immunized IRF3^−/−^ and IFNAR^−/−^ mice was simply due to an increase in LS parasite biomass and thus antigen load when compared to B6 mice. Using a bioluminescent LARC GAP for immunization, we observed no significant differences in LS parasite burden or persistence (Supplementary Fig. 2A). Results were confirmed by quantitative reverse transcription (qRT) PCR (Supplementary Fig. 2B). As such, we conclude that the observed enhancement in protective efficacy (Fig. 1) in IRF3^−/−^ and IFNAR^−/−^ mice immunized with LARC GAP is not due to increased parasite biomass and antigen load in the absence of IFN-1 signaling.

Together, the data derived from two distinct RC whole sporozoite immunization strategies clearly indicate that the innate IFN-1 response curtails the generation of optimal adaptive protective immunity. This, however, does not occur with RD parasite immunization.

### Hepatic CD8 T cells mediate LARC GAP protection

Protection after immunization with whole sporozoites is critically dependent on the generation of memory CD8 T cell responses^19^. Moreover, we have previously shown that B6 mice immunized with LARC GAP and depleted of CD8 T cells at the time of challenge completely lose sterile protection^15^. We here confirmed the importance of CD8 T cells in LARC-mediated protection (Supplementary Fig. 3A, B) but also wanted to ascertain in which anatomical site these critically protective memory CD8 T cells are generated and might reside. To this end, we repeated the immunization-challenge in splenectomized mice and control mice that had undergone a sham splenectomy (Supplementary Fig. 3C). Mice that had undergone a splenectomy were completely protected against sporozoite challenge as were control mice (Supplementary Fig. 3D). Thus, while CD8 T cells are critical for protection, the spleen is not required for their development nor does it serve as an important reservoir of protective memory CD8 T cell responses after LARC GAP immunization.

**Fig. 2:**
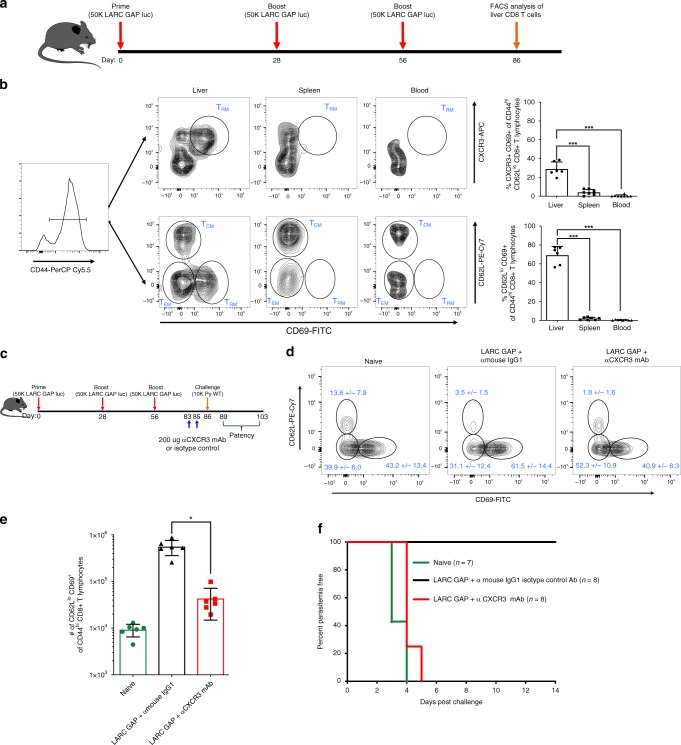
Liver memory CD8 T cells generated in LARC GAP-immunized mice are critical to protection. **a** Schematic of the immunization regimen. Mice were immunized thrice with 50,000 LARC GAP sporozoites prior to memory CD8 T cell characterization by FACS analysis. **b** Gating strategy outlining the characterization of memory CD8 T cells in the three anatomical sites examined. Memory CD8 T cells are stratified either as CD44^hi^CXCR3+CD69+ of CD62Llo CD8+ T lymphocytes or CD44hiCD62L^lo^CD69+ of CD8+ T lymphocytes. **c** Schematic of immunization with CXCR3 depletion. Mice were immunized thrice with 50,000 LARC GAP sporozoites and then treated with a CXCR3-depleting mAb twice before a sporozoite challenge. **d** Injection with a CXCR3 mAb reduces the proportion of liver memory CD8 T cells. **e** Injection with a CXCR3 mAb reduces the total number of liver memory CD8 T cells in immunized mice. **f** Examination of blood stage infection in immunized B6 mice treated with a CXCR3-depleting mAb or isotype control antibody and challenged with WT sporozoites 30 days post last immunization. Injection with a CXCR3 mAb abrogates protection against a sporozoite challenge. Data from panels **b**, **d**–**f** are compiled from two independent experiments with at least three mice in each group per experiment. Each dot represents a single mouse. Bar graphs are expressed as mean ±  SD. Total number of mice in each experiment is shown in the survival curves. ***p < 0.0001, *p < 0.05 (from unpaired two-tailed Student’s *t*-test). Components of this figure were created using Servier Medical Art templates, which are licensed under a Creative Commons Attribution 3.0 Unported License; https://smart.servier.com

Recent studies indicate that liver-resident memory CD8 T cells (T_RM_) form the basis of cell-mediated protection after immunization with RAS^17,18,28,29^. However, the contribution of liver T_RM_s to LARC GAP-engendered protection has not been documented. Thus, we immunized B6 mice three times with LARC GAP and examined the proportion of putative T_RM_s in different anatomical sites (liver, spleen, and blood) 30 days after the last immunization (Fig. 2a). We characterized putative T_RM_s as CD44^hi^ CXCR3^+^CD69^+^ or CD44^hi^CD62L^lo^CD69^+^ (Fig. 2b, left panel) as has been reported for RAS-immunized mice^17,30^. We observed robust populations of these memory CD8 T cells in the liver but not the spleens or blood of LARC GAP-immunized mice (Fig. 2b, quantified in right panel). To determine if the CXCR3-expressing cells were critical for protection, we immunized B6 mice three times with LARC GAP and then depleted CXCR3-expressing cells shortly before challenge (Fig. 2c). We observed a reduction in the proportion (Fig. 2d) and the absolute numbers (Fig. 2e) of CD44^hi^CD62L^lo^CD69^+^ CD8 T cells in the livers of immunized mice that received the CXCR3 monoclonal antibody as compared to mice that received an isotype control antibody. Moreover, while mice that received the isotype control antibody were completely protected, mice that received the CXCR3-depleting antibody lost sterile protection (Fig. 2f).

Thus, we conclude that CD8 T cells and in particular, CD69+ memory CD8 T cells in the liver are essential for the protective adaptive immune response engendered by LARC GAP immunization.

### IFNAR^−/−^ mice generate greater hepatic CD8 T cell memory

**Fig. 3:**
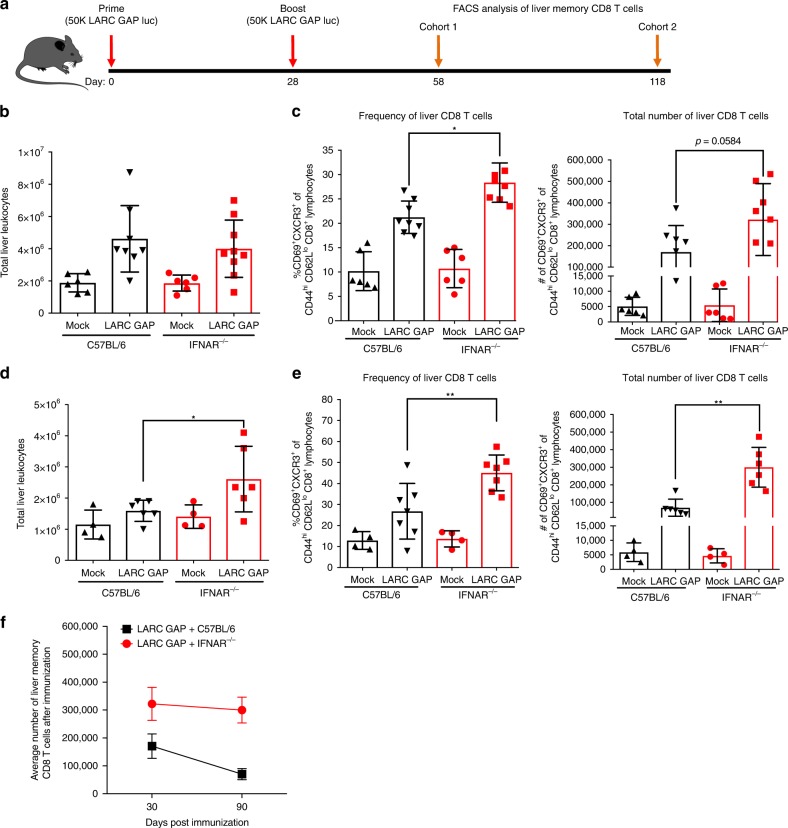
Lack of type I IFN signaling enhances the generation of liver memory CD8 T cells in LARC GAP-immunized mice. **a** Schematic of the immunization regimen. B6 mice and IFNAR^−/−^ mice were immunized twice with 50,000 LARC GAP sporozoites. Thirty or 90 days after the second immunization liver non-parenchymal cells were isolated and subjected to FACS analysis. **b** Quantification of liver leukocytes isolated from immunized B6 mice and immunized IFNAR^−/−^ mice, 30 days after the last immunization. **c** Analysis of liver memory CD8 T cells 30 days after GAP immunization. Liver memory CD8 T cells are defined as CD44^hi^CXCR3+CD69+ of CD62Llo CD8+CD4−CD3+ T lymphocytes. **d** Quantification of liver leukocytes isolated from immunized B6 mice and immunized IFNAR^−/−^ mice, 90 days after the last immunization. **e** Analysis of liver memory CD8 T cells 90 days after immunization. Liver memory CD8 T cells are defined as CD44^hi^CXCR3+CD69+ of CD62Llo CD8+CD4−CD3+ T lymphocytes. **f** Comparison of the mean liver memory CD8 T cell numbers 30 or 90 days after boost. Data from panels **b** through **f** are compiled from at least two independent experiments with two mock immunized and at least three LARC GAP immunized mice in each group. Each dot represents a single mouse. Bar graphs are expressed as mean ± SD. *p < 0.05 and **p < 0.005 (from unpaired two-tailed Student’s *t*-test). Components of this figure were created using Servier Medical Art templates, which are licensed under a Creative Commons Attribution 3.0 Unported License; https://smart.servier.com

Given our observation that memory CD8 T cells in the liver are critical for protection by LARC GAP, we examined the impact of IFN-1 signaling on the memory CD8 T cell population in the livers of B6 and IFNAR^−/−^ mice 30 days after the last LARC GAP immunization (Fig. 3a). Of note, this time point was chosen since we have shown enhanced protection in immunized IFN-1 signaling-deficient mice when the sporozoite challenge was given 30 days after the last immunization (Fig. 1b, c). We observed no significant difference in the total number of leukocytes (Fig. 3b) in the livers of immunized IFNAR^−/−^ mice as compared to immunized B6 mice. However, upon stratification of the CD8 T cell population into the protective CD44^hi^CD69^+^CXCR3^+^ memory CD8 T cell population^17^, we observed a greater proportion of these cells in immunized IFNAR^−/−^ mice as compared to immunized B6 mice (Fig. 3c, left panel). We also observed an increase in the total number of CD44^hi^CD69^+^CXCR3^+^ memory CD8 T cells in immunized IFNAR^−/−^ mice although this did not reach statistical significance (Fig. 3c, right panel). We have observed that in contrast to two-dose LARC GAP-immunized B6 mice, the majority of two-dose LARC GAP immunized IFNAR^−/−^ mice maintain complete sterile protection at 90 days (Fig. 1e). To examine whether this correlates with greater memory CD8 T cell responses, we immunized cohorts of B6 and IFNAR^−/−^ mice, harvested the livers from each mouse, and characterized the liver memory CD8 T cell population 90 days after the last immunization by flow cytometry (Fig. 3a). The livers of LARC GAP-immunized IFNAR^−/−^ mice contained nearly 1.6 times more liver leukocytes (Fig. 3d), a greater proportion of liver CD44^hi^CD62L^lo^CD69^+^CXCR3^+^ memory CD8 T cells (Fig. 3e, left panel), and greater total numbers of liver CD44^hi^CD62L^lo^CD69^+^CXCR3^+^ memory CD8 T cells (Fig. 3e, right panel) than the immunized B6 mice. Indeed, by plotting the average number of CD44^hi^CD62L^lo^CD69^+^CXCR3^+^ memory CD8 T cells in the livers of immunized B6 or IFNAR^−/−^ mice we observed that while liver CD44^hi^CD62L^lo^CD69^+^CXCR3^+^ memory CD8 T cell numbers are significantly reduced in B6 mice between day 30 and day 90 post immunization, there is a slower attrition of CD44^hi^CD62L^lo^CD69^+^CXCR3^+^ memory CD8 T cells in the livers of immunized IFNAR^−/−^ mice (Fig. 3f).

**Fig. 4:**
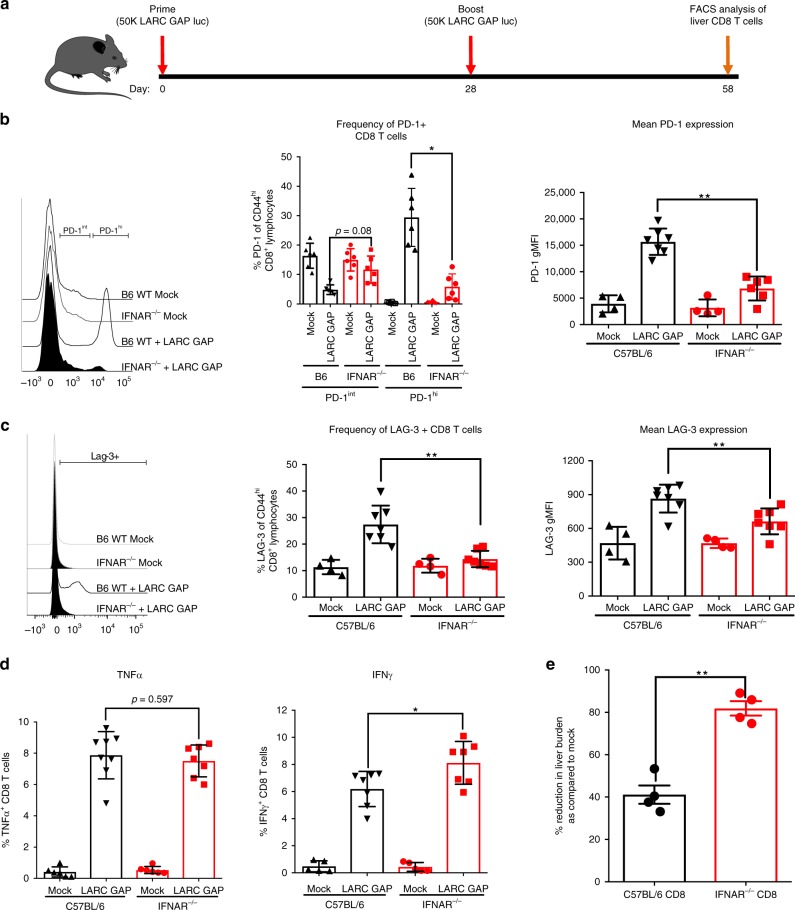
Type I IFN signaling increases CD8 T cell exhaustion and memory T cell turnover in LARC GAP-immunized mice. **a** Schematic of the immunization regimen. B6 mice and IFNAR^−/−^ mice were immunized twice with 50,000 LARC GAP sporozoites. Thirty days after the second immunization, liver leukocytes cells were isolated and subjected to FACS analysis. **b** Expression of the co-inhibitory marker PD-1 is reduced on liver CD8 T cells isolated from immunized IFNAR^−/−^ mice when compared to immunized WT B6 mice. PD-1+ cells are defined as PD-1^hi/int^CD44^hi^ of CD8+CD4−CD3+ T lymphocytes. **c** Expression of the co-inhibitory marker LAG-3 is reduced on liver CD8 T cells isolated from immunized IFNAR^−/−^ mice when compared to immunized B6 mice. LAG-3+ cells are defined as LAG-3+CD44^hi^ of CD8+CD4−CD3+ T lymphocytes. **d** FACS analysis of ex vivo-stimulated liver CD8 T cells isolated from immunized B6 mice and immunized IFNAR^−/−^ mice. The proportion of TNFα and IFNγ was quantified in enriched CD8 T cells. **e** Adoptive transfer of 2.5 × 10^6^ liver CD8 T cells isolated from immunized B6 or immunized IFNAR^−/−^ mice and subsequently transferred into naïve B6 mice prior to challenge with 1500 *Py* GFPluc sporozoites. Data from panels **b** and **c** are compiled from two independent experiments with two mock immunized and three LARC GAP immunized mice in each group. Bar graphs are expressed as mean ± SD. Data from panels **d** and **e** represent one of two independent experiments. Each dot represents a single mouse. *p < 0.05 and **p < 0.005 (from unpaired two-tailed Student’s *t*-test). Components of this figure were created using Servier Medical Art templates, which are licensed under a Creative Commons Attribution 3.0 Unported License; https://smart.servier.com

The increased attrition of CD44^hi^CD62L^lo^CD69^+^CXCR3^+^ memory CD8 T cells in the livers of immunized B6 mice is likely due to decreased survival signaling in memory CD8 T cells generated in the presence of LS parasite-induced IFN-1 signaling at the time of immunization. Interestingly, in models of chronic viral infection, sustained and/or elevated IFN-1 signaling has been implicated in the induction of a dysfunctional state in the memory T cell response that is characterized by increased susceptibility to cell death, impaired cytokine production, reduced proliferation, and reduced effector function^31,32^. This “exhausted” or “dysfunctional” state is characterized by the increased expression of co-inhibitory molecules including programmed cell death protein 1 (PD-1), lymphocyte activation gene 3 (LAG-3), and cytotoxic T lymphocyte antigen 4 (CTLA-4) on the surface of memory T cells^32^. As such, we evaluated the impact of IFN-1 signaling on the quality of the memory CD8 T cell response by examining the expression of two of these inhibitory molecules, PD-1 and LAG-3 (ref. ^32^) on memory CD8 T cells isolated from the livers of LARC GAP-immunized B6 and IFNAR^−/−^ mice (Fig. 4a). The proportion of PD-1^hi^CD44^hi^CD8^+^ T cells was reduced in immunized IFNAR^−/−^ mice (Fig. 4b, top and middle panels). Additionally, PD-1 expression was also reduced on individual CD8 T cells isolated from the livers of immunized IFNAR^−/−^ mice (Fig. 4b, bottom panel). In agreement with the PD-1 data, we also observed fewer LAG-3-expressing CD8 T cells (LAG-3^+^CD44^hi^CD8^+^) (Fig. 4c, top and middle panels) as well as reduced LAG-3 expression on CD8 T cells isolated from the livers of immunized IFNAR^−/−^ mice (Fig. 4c, bottom panel).

Thus, without IFN-1 signaling, immunized mice generated greater numbers of liver memory CD8 T cells and these cells showed reduced expression of co-inhibitory molecules at the memory time point.

### IFNAR^−/−^ CD8 T cells display superior effector function

We identified greater proportions and absolute numbers of liver memory CD8 T cells in LARC GAP-immunized IFNAR^−/−^ mice. Moreover, given the reduced PD-1 and LAG-3 expression on liver memory CD8 T cells isolated from immunized IFNAR^−/−^ mice, we predicted that these cells would retain improved effector responses upon secondary antigen exposure. CD8 T cell elimination of LS infection is dependent, at least in part, on the production of TNFα and IFNγ^33^. We thus set out to determine whether CD8 T cells isolated from LARC GAP-immunized IFNAR^−/−^ mice produced higher levels of these cytokines upon re-stimulation compared to B6 immunized mice. B6 and IFNAR^−/−^ mice were immunized with LARC GAP as described in Fig. 1a. Four weeks after the last immunization, we isolated liver non-parenchymal cells, enriched for CD8 T cells by negative selection and then stimulated equivalent numbers of CD8 T cells with plate-bound anti-CD3 and anti-CD28 for 6h to examine if there were any differences in the effector functions of memory CD8 T cells generated in the presence or absence of IFN-1 signaling. We did not observe any differences in the proportion of TNFα producing CD8 T cells (Fig. 4d, top panel), but did observe that a greater proportion of the CD8 T cells isolated from immunized IFNAR^−/−^ mice produced IFNγ (Fig. 4d, bottom panel). Given the higher proportion of IFNγ-producing CD8 T cells derived from immunized IFNAR^−/−^ mice, we next set out to determine if these CD8 T cells would more effectively eliminate LS-infected hepatocytes in vivo when compared to CD8 T cells from immunized B6 mice. To this end, we adoptively transferred 2.5 × 10^6^ CD8 T cells isolated from the livers of LARC GAP-immunized IFNAR^−/−^ and B6 mice into naïve B6 mice and challenged each mouse 24 h later with 1.5 × 10^3^ *Py* GFPluc sporozoites. After 44 h, we measured LS parasite burden by in vivo bioluminescence imaging. Adoptively transferred CD8 T cells from immunized B6 mice reduced LS burden by 40%, while transfer of CD8 T cells from immunized IFNAR^−/−^ mice led to an 80% reduction in LS burden, demonstrating superior effector function of memory CD8 T cells derived from an environment lacking IFN-1 signaling (Fig. 4e). Together with the increased memory CD8 T cell attrition, the observation of impaired effector responses supports the notion that LS-induced IFN-1 signaling impairs protection in part through the generation of dysfunctional memory T cells.

### Hepatic T cell expansion is not enhanced in IFNAR^−/−^ mice

**Fig. 5:**
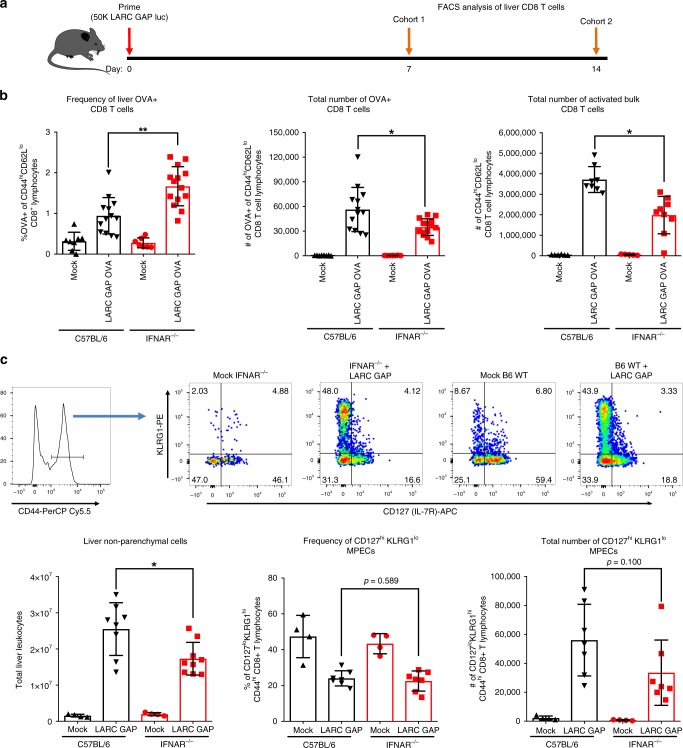
CD8 T cell priming, expansion, and generation of long-lived memory precursors is not enhanced in LARC GAP immunized IFNAR^−/−^ mice. **a** Schematic of the immunization regimen. B6 mice and IFNAR^−/−^ mice were immunized once with 50,000 LARC GAP or LARC GAP OVA sporozoites and liver lymphocyte populations analyzed either at day 7 or day 14 post immunization. **b** Analysis of the proportion (left panel) and total number (middle) of OVA-specific and bulk activated (right panel) liver CD8 T cells, 7 days after immunization. OVA-specific CD8 T cells are characterized as OVA+ of CD44hiCD62Llo CD8+CD4−CD3+ T lymphocytes. Bulk activated CD8 T cells are characterized as CD44^hi^CD62L^lo^ CD8+CD4−CD3+ T lymphocytes. **c** Analysis of short-lived effectors (SLEC) and long-lived memory precursor effector cells (MPEC) at day 14 post primary immunization. Gating strategy showing KLRG1^hi^CD127^lo^ SLEC and KLRG1^lo^CD127^hi^ MPEC CD8 T cells in the livers of immunized mice (top panel). Quantification of the total number of liver leukocytes (bottom, left panel), proportion of MPECs (bottom, middle panel), and total number of MPECs (bottom, right panel). Data are compiled from two independent experiments with two mock immunized and three LARC GAP immunized mice in each group. Each dot represents an individual mouse. Bar graphs are expressed as mean ± SD. *p < 0.05, and **p < 0.005 (from unpaired two-tailed Student’s *t*-test). Components of this figure were created using Servier Medical Art templates, which are licensed under a Creative Commons Attribution 3.0 Unported License; https://smart.servier.com

We observed greater numbers of memory CD8 T cells in the livers of immunized IFNAR^−/−^ mice 30 days after the last immunization, implying that more liver memory CD8 T cells might be generated in these mice during priming. To determine if lack of IFN-1 signaling impacts the initial education of antigen-specific CD8 T cells, we immunized B6 and IFNAR^−/−^ mice with a LARC GAP that expresses the chicken ovalbumin protein (LARC GAP OVA) and examined liver CD8 T cell responses 7 days later (Fig. 5a) using the H-2k^b^ restricted MHC class I peptide tetramer (SIINFEKL/K^b^). The proportion of OVA-specific CD8 T cells (OVA^+^CD44^hi^CD62L^lo^) was higher in the livers of immunized IFNAR^−/−^ mice as compared to immunized B6 mice (Fig. 5b, left panel). However, we isolated over two times as many leukocytes from the livers of B6 mice. Thus, upon enumeration of the absolute number of OVA-specific CD8 T cells, we observed slightly more OVA-specific CD8 T cells in the livers of immunized B6 mice (Fig. 5b, middle panel). In agreement with this observation, the absolute number of activated bulk liver CD8 T cells was higher in immunized B6 mice as compared to immunized IFNAR^−/−^ mice (Fig. 5b, right panel). Thus, while loss of IFN-1 signaling leads to a significant increase in the number of memory CD8 T cells in the liver at late timepoints after immunization (day 30 and day 90), this is not due to increased priming and expansion of a greater number of effector CD8 T cells. The duration and magnitude of inflammatory signaling can also dictate the proportion of effector cells that commit to long-term memory development^34^. In particular, high inflammatory signaling can tilt the balance of T cell education towards the production of short-lived effector cells instead of long-lived memory precursors. Thus, we immunized B6 and IFNAR^−/−^ mice with LARC GAP and examined the population of long-lived memory precursor effector cells (MPECs) as defined by increased CD127 expression and reduced KLRG1 expression (KLRG1^lo^CD127^hi^ CD44^hi^ CD8+ T cells) (gating strategy in Fig. 5c, top panel). We observed nearly two-fold more liver leukocytes in immunized B6 mice as compared to their IFNAR^−/−^ counterparts (Fig. 5c, bottom left panel). Moreover, both the proportion (Fig. 5c, bottom middle panel) and the total number (Fig. 5c, bottom right panel) of MPECs was not significantly enhanced in immunized IFNAR^−/−^ mice. Thus, the greater number of liver memory CD8 T cells in immunized IFNAR^−/−^ mice at late timepoints does not stem from the generation of larger numbers of long-lived memory precursors in the absence of IFN-1 signaling.

### IL-7Rα is increased on hepatic CD8 T cells of IFNAR^−/−^ mice

If neither the expansion of effector CD8 T cells nor their commitment to long-lived memory precursors is enhanced during priming, then what accounts for the increase in liver memory CD8 T cells observed late after immunization? We observed a greater attrition of liver memory CD8 T cells generated in the presence of LS parasite-induced IFN-1 signaling (Fig. 3f). We thus postulated that this increased attrition of memory CD8 T cells in the livers of immunized B6 mice was likely due to decreased survival signaling or increased death signaling in memory CD8 T cells generated in the presence of LS parasite-induced IFN-1 signaling. As such, we immunized B6 and IFNAR^−/−^ mice once with LARC GAP and examined the quality of the memory CD8 T cell response in the liver 30 days later (Supplementary Fig. 4A). We observed reduced expression of PD-1 (Supplementary Fig. 4B) and LAG-3 (Supplementary Fig. 4C) on memory CD8 T cells isolated from the livers of IFNAR^−/−^ mice. Additionally, we also examined the expression of IL-7Rα (CD127), a receptor critical for the survival and homeostatic proliferation of memory CD8 T cells^35^, on CD8 T cells isolated from the livers of immunized mice. We observed higher IL-7Rα expression on liver CD8 T cells isolated from LARC GAP-primed IFNAR^−/−^ mice (Supplementary Fig. 4D). Thus, in the presence of competent IFN-1 signaling, LARC GAP immunization results in the expression of markers indicative of impaired memory T cell survival and increased T cell dysfunction. We propose that this impairment in T cell survival signaling likely contributes to the decreased numbers of memory CD8 T cells observed late after immunization.

### Impaired CD8 T cell memory is mediated by CD8 T cell-extrinsic IFNAR

**Fig. 6:**
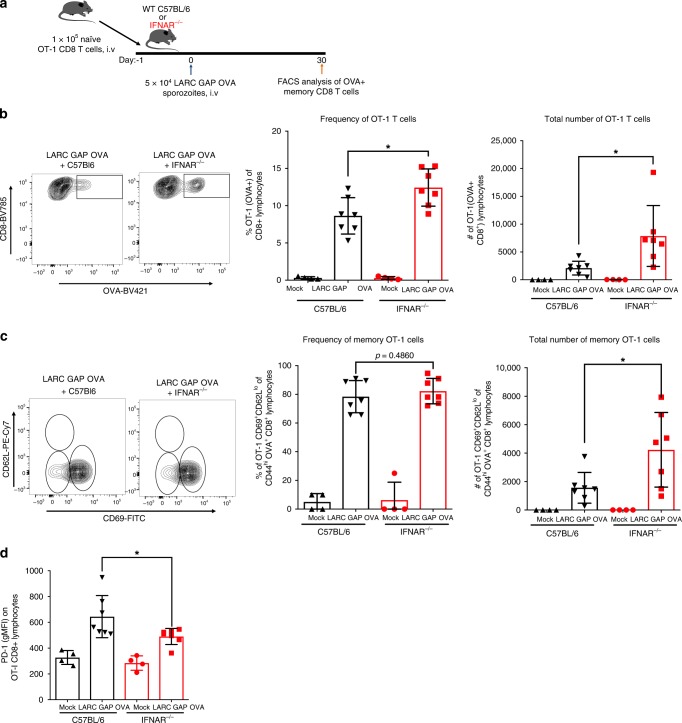
The detrimental role of the parasite-engendered type I IFN response is extrinsic to the CD8 T cell compartment. **a** Schematic of OT-1 adoptive transfer and subsequent immunization regimen. WT B6 mice and IFNAR^−/−^ mice received 100,000 naïve OT-1 cells 24 h prior to immunization with 50,000 LARC GAP OVA sporozoites. Thirty days later, liver non-parenchymal cells were isolated and subjected to FACS analysis. **b** Quantification of OT-1 CD8 T cells by FACS analysis. OT-1 T cells were identified using the SIINFEKL MHC-I restricted OVA tetramer. **c** Analysis of OT-1 liver memory CD8 T cells 30 days after GAP immunization. liver memory CD8 T cells are defined as CD69+CD62L^lo^ of CD44^hi^OVA+CD8+CD4−CD3+T lymphocytes. **d** Analysis of PD-1 expression on OT-1 memory CD8 T cells. PD-1+ cells are defined as PD-1+ of CD44^hi^OVA+CD8+CD4−CD3+ T lymphocytes. Data from panels **b** through **d** are compiled from two independent experiments with two mock immunized and three GAP immunized mice in each group. Each dot represents a single mouse. Bar graphs are expressed as mean ± SD. *p < 0.05 (from unpaired two-tailed Student’s *t*-test). Components of this figure were created using Servier Medical Art templates, which are licensed under a Creative Commons Attribution 3.0 Unported License; https://smart.servier.com

We next asked whether the enhanced memory CD8 T cell response in IFNAR^−/−^ mice is due to a lack of IFNAR signaling on CD8 T cells or rather due to a lack of IFNAR on other cells in the environment within which these CD8 T cells are educated. To address this, we isolated 100,000 naïve WT OT-1 CD8 T cells from the spleens of transgenic mice in which the CD8 T cell receptor has been engineered to recognize the processed SIINFEKL peptide on MHC-I. These naïve OT-1 CD8 T cells were then transferred into naïve B6 or IFNAR^−/−^ mice 24 h prior to immunization with the OVA expressing LARC GAP. We then examined liver memory T cell responses in the adoptively transferred OT-1 cell population 30 days later (Fig. 6a). We observed a greater proportion of OT-1 CD8 T cells (Fig. 6b, left and middle panels) and greater total numbers of OT-1 CD8 T cells (Fig. 6b, right panel) in the livers of immunized IFNAR^−/−^ mice. Moreover, while we did not observe a significant difference in the proportion of OVA-specific CD44^hi^CD62L^lo^CD69^+^CXCR3^+^ memory CD8 T cells within the livers of immunized IFNAR^−/−^ mice (Fig. 6c, left and middle panels), the total number of OVA-specific CD44^hi^CD62L^lo^CD69^+^CXCR3^+^ memory CD8 T cells in IFNAR^−/−^ mice was significantly increased by nearly twofold as compared to immunized B6 mice (Fig. 6c, right panel). Lastly, we also observed a reduction in the expression of PD-1 on memory OT-1 T cells generated in IFNAR^−/−^ mice (Fig. 6d). Thus, we conclude that the detrimental role for the parasite-engendered IFN-1 signaling cascade is due to IFN-1 signaling in a cell type other than the CD8 T cell.

## Discussion

Efforts to develop highly efficacious malaria vaccines have been buoyed by the the use of whole parasites as immunogens^4,5^. Replication-competent parasites that unfold their full antigenic potential in the liver are superior to replication-deficient parasites that arrest early in liver infection. LARC GAPs likely constitute the most potent whole sporozoite immunogen given that in mouse models, they afford superior protection as compared to RD RAS and EARD GAPs^7^, and engender stage and species-transcending protection^7,8^. LARC parasite vaccination also stimulates an innate IFN-1 response and we show here that surprisingly, this negatively impacts the acquisition of protective adaptive immunity, and thus ablation of IFN-1 signaling enhances protection. Importantly, the enhanced protection against a sporozoite challenge in IFN-1-deficient mice correlated with an increase in the magnitude and quality of memory CD8 T cells in the liver. The impaired quality of the memory response in the presence of IFN-1 signaling was characterized by the increased expression of the T cell co-inhibitory molecules PD-1 and LAG-3 as well as reduced expression of the pro-survival IL-7Rα on memory CD8 T cells isolated from IFN-1 signaling competent hosts.

Innate immunity serves as the first line of defense against invading pathogens and is also instrumental for stimulating and shaping the ensuing adaptive immune response. Type I IFNs have well documented protective roles against multiple pathogens and have been targeted to boost the adaptive immune response to a variety of vaccines^22–24^. However, recent studies have also  identified unexpected negative roles for IFN-1 in that they impair optimal adaptive immunity and enhance disease progression^24,31,36–39^. Immunoregulatory mechanisms that impair adaptive immunity are induced upon persistent, systemically elevated IFN-1 signaling in chronic viral^31,36^ and parasitic infections including blood stage malaria^38,39^. In contrast to chronic blood stage malaria infection, the pre-erythrocytic stage infection investigated herein is transient and short-lived (2 days for rodent malaria parasites and 6–10 days for human parasites) and attenuated parasites do not persist in the liver^40^. Additionally, studies from our laboratory and others indicate that pre-erythrocytic infection does not result in a systemic, hyper inflammatory IFN-1 response as has been observed during acute infections with other pathogens^20,26^. Indeed the LS infection-induced IFN-1 protein is only detected in liver tissue implying that the IFN-1 mediated inflammatory response is restricted to the liver^20,26^. Thus, it is surprising that this limited hepatic IFN-1 response would have such a durable negative effect on the ensuing adaptive immune response.

Mouse models of *Plasmodium* infection strongly support a critical role for CD8 T cells as mediators of protective adaptive immune responses generated by whole sporozoite immunization^19,41^. Additionally, with the identification of resident, non-recirculating memory CD8 T cell populations in organs such as the skin and gut^42^, it was postulated that resident memory CD8 T cells in the liver would be critical for pre-erythrocytic immunity against infected hepatocytes. In mice immunized with RAS, liver resident memory CD8 T cells express distinct markers including CXCR3, CXCR6, and CD69, which are not expressed on effector memory CD8 T cells in the liver or memory CD8 T cells isolated from other anatomical sites. While these liver T_RM_s are blood accessible, they do not recirculate but rather patrol liver sinusoids to survey for infected hepatocytes^17,18,28^. Here, we report that LARC GAP immunization also induces a population of memory CD8 T cells that express these aforementioned T_RM_ markers (i.e CXCR3 and CD69). Moreover, similar to studies in RAS-immunized mice, we also report that the LARC GAP immunization induced T_RM_ population is largely absent in the blood and spleen. Importantly, the protective immune response attributed to LARC GAP immunization is eliminated upon injection of mice with a CXCR3-depleting antibody. While this circumstantial evidence indicates that these memory CD8 T cells are liver resident memory CD8 T cells (i.e T_RM_s), in the absence of functional evidence of true liver residency we cannot formally claim that the memory CD8 T cells identified in the livers of LARC GAP-immunized mice are bonafide T_RM_s. Nonetheless, we show that LARC GAP-immunized IFNAR^−/−^ mice are better protected against a sporozoite challenge and generated significantly more memory liver CD8 T cells as compared to immunized B6 mice, yet  the increase in memory liver CD8 T cells in immunized IFNAR^−/−^ mice did not stem from a greater expansion of effector CD8 T cells. We then thought that the reduced numbers of liver memory CD8 T cells in immunized B6 mice might be driven by an increased generation of terminal short-lived effector cells as compared to long-lived memory CD8 T cells in the presence of competent IFN-1 signaling. Counter to this notion, we actually observed reduced numbers of long-lived memory precursor effector cells in immunized IFNAR^−/−^ mice.

So, what accounts for the larger numbers of memory CD8 T cells in the livers of IFNAR^−/−^ mice? Memory CD8 T cells isolated from immunized IFNAR^−/−^ mice are of better quality as evidenced by reduced expression of the exhaustion markers PD-1 and LAG-3. T cell exhaustion is typified by impairments in memory T cell survival, as well as cytokine production, proliferation, and effector function upon antigen re-encounter^32^. In support of this, we show that CD8 T cells isolated from the livers of immunized IFNAR^−/−^ mice more readily produce IFNγ and upon adoptive transfer into naïve mice, eliminated LS-infected hepatocytes more effectively. A key feature of memory T cells is the development of antigen-independent self-renewal, a property that is critical to the long-term survival of memory T cells in the host. This homeostatic survival is in part driven by engagement of IL-7 with its cognate receptor IL-7Rα (CD127)^34,43,44^. We show that although numbers of liver memory CD8 T cells rapidly decline in B6 mice between day 30 and day 90 post LARC GAP immunization, this decline is less pronounced in immunized IFNAR^−/−^ animals. Coinciding with this, we observed higher IL-7Rα expression on memory CD8 T cells isolated from IFNAR^−/−^ mice. In chronic viral infections, T cell exhaustion correlates with reduced expression of IL-7Rα resulting in the poor maintenance of memory T cells^32^. Together, these data strongly indicate that the LS-engendered IFN-1 signaling cascade drives a dysfunctional T cell program. This dysfunctional state results in an impairment of effector CD8 T cell function and enhanced T cell death that likely explains the reduced liver memory CD8 T cell numbers and inability to effectively confer protection when suboptimally immunized B6 mice were challenged.

Pathogenic CD8 T cell intrinsic and extrinsic IFN-1 signaling have been reported in blood stage *Plasmodium* infection^38,45^. In our study we utilized the adoptive transfer of naïve OT-I CD8 T cells into B6 or IFNAR^−/−^ mice to show that the detrimental role of IFN-1 signaling after LARC GAP OVA immunization is not due to IFNAR expression on CD8 T cells. In chronic infections, conventional dendritic cells upregulate the PD-1 ligands PD-L1 and PD-L2 and produce immunoregulatory cytokines in response to IFN-1 signaling, all factors that can promote T cell dysfunction^38,46^. LS-engendered IFN-1 signaling induces the recruitment of multiple leukocytes into the liver^20,26^. Indeed, a recent study indicates that monocyte-derived dendritic cells recruited into the liver acquire *Plasmodium* antigen from infected hepatocytes to prime CD8 T cells^47^. Additionally, during blood stage *Plasmodium* infection, IRF3 expression modulates chemokine production and MHC-II expression in inflammatory monocytes^48^. As such, it is tempting to speculate that IFN-1 signaling in myeloid cell populations detrimentally impacts the generation of protective pre-erythrocytic adaptive T cell immunity.

Together, our findings have uncovered a detrimental role of the innate immune response in development of optimal protective adaptive immunity against a transient hepatotropic pathogen. This might inform new strategies to further improve upon the efficacy of whole parasite malaria vaccines that aim at preventing infection.

## Methods

### Ethics statement

Animal studies were performed according to the regulations of the institutional animal care and use committee. Approval was obtained from the Seattle Children’s Research Institute Instituitional Animal Care and Use Committee (IACUC) under protocol 00507. The Seattle Children’s Research Institute IACUC adheres to the NIH Office of Laboratory Animal Welfare standards (OLAW welfare assurance # D16-00119).

### Mice

Female Swiss Webster (SW) mice for parasite cycles were purchased from Envigo laboratories. Six- to 8-week-old female C57BL/6 mice were purchased from Jackson Laboratories. IRF3^−/−^ and IFNAR1^−/−^ on the C57BL/6 background were kindly provided by Dr. Michael Gale at the University of Washington. Six- to 8-week-old OT-I (C57BL/6-Tg (TcraTcrb)1100Mjb/J) mice were purchased from Jackson Laboratories. Mice were maintained and bred under specific pathogen-free conditions at the Center for Global Infectious Disease Research, Seattle Children’s Research Institute.

### Sporozoite isolation

Six- to 8-week-old SW mice were injected intraperitoneally (i.p.) with blood stage *Py* GFPluc^27^ and *Py fabb/f*^–^^25^ expressing luciferase or the chicken ovalbumin antigen. Three days later, gametocyte exflagellation was confirmed and the infected mice were used to feed female *Anopheles stephensi* mosquitoes. Fourteen to 16 days after the feed, salivary gland sporozoites were isolated from the mosquitoes and used in mouse infections.

### Mouse immunizations and challenges

Age matched, 8- to 12-week-old female WT C57BL/6, IRF3^−/−^ or IFNAR1^−/−^ mice were immunized intravenously (i.v.) either via tail vein or retro-orbital plexus injection with 50,000 sporozoites or as a control with salivary gland debris from uninfected mosquitos. For immunizations under drug cover, immunized mice were also treated twice (30 and 40 hpi) with 14.4 mg/kg of Atovaquone. For challenge experiments, immunized mice were challenged intravenously 4–5 weeks after the last immunization with 10,000 *Py* WT or 10,000 *Py* GFPluc sporozoites. Parasite development in the liver was determined by in vivo bioluminescent imaging^27^ or qRT PCR. Parasitemia was monitored by Giemsa-stained thin smears beginning on day three post challenge. At least 20 fields of >200 red blood cells per field were examined for patency determination. Mice showing no evidence of blood stage infection by day 14 were assessed as sterilely protected.

### Organ harvests, cell isolation, and flow cytometry

For the analysis of liver memory CD8 T cells 4 to 5 weeks after immunization, livers of immunized mice harvested 4 weeks after the last immunization were cut into small pieces, mechanically disrupted using the plunger of a 10 mL syringe and strained through a 100 µm nylon filter. The non-parenchymal cell fraction was isolated as outlined previously^27^. For the analysis of liver non-parenchymal cells at day 7 and day 14, the liver was perfused and digested with collagenase as previously described^49^. For the phenotypic analysis of splenic lymphocytes, mouse spleens were harvested and homogenized to form single cells suspensions of splenocytes. Red blood cells were removed by lysis with ACK lysis buffer and the splenocytes strained through a 100 µm nylon filter. For the analysis of peripheral memory CD8 T cells, heparinized blood was collected from immunized mice and treated with ACK lysis buffer to remove red blood cells. Isolated cells were incubated with mouse Fc block (#553141 BD Biosciences) for 20 min at 4C and the stained with surface antibodies specific for CD3 (0.7:100, clone 145-2C11, #100222 or #100351), CD4 (0.7:100, clone GK1.5,#100548), CD8 (0.7:100, clone 536.7, #100749), CD19 (0.7:100, clone 6D5, #115541), CD44 (1:200, clone IM7, # 103032), CD62L (1:200, clone MEL-14, #104417), CD69 (1:100, clone HI.2F3, #104506), PD-1/CD279 (1:100, clone 29F.1A12, #135217 or #135210), CD223/LAG-3 (1:100, clone C9B7W, #125208 or #125219), CD127/IL-7Rα (1:100, clone A7R34, #135012), KLRG1(1:100, clone 2F1, #138407), and CXCR3 (1:50, clone CXCR3-173, #4338627). OVA-specific CD8 T cells were identified using an MHC-I tetramer against the H-2k^b^ peptide SIINFEKL (NIH tetramer core). Labeled cells were run on an LSRII flow cytometer (BD Biosciences, Franklin lakes NJ) and analyzed with FlowJo software (Tree Star).

### Depletion of liver memory CD8 T cells

To deplete liver CD8+ T cells, 0.5 mg of anti-CD8 mAb 2.43 (BioXCell) or 0.5 mg of isotype control rat IgG (BioXCell) was injected into immunized C57BL/6 WT mice via the retro-orbital plexus 24 h prior to sporozoite challenge. CD8 T cell depletion was confirmed before each challenge by collecting 100 μL of peripheral blood via the retro-orbital plexus from each mouse and assaying peripheral blood lymphocytes by flow cytometry after staining cells with mAb against CD3 (0.7:100, clone 145-2C11, #100222), CD4 (0.7:100, clone GK1.5, #100548), CD8 (0.7:100, clone 536.7, #100749), CD19 (0.7:100, clone 6D5, #115541). To deplete liver memory CD8 T cells, LARC-GAP immunized mice were intravenously injected with two doses (200  and 100 μg) of anti-CXCR3 antibody or the isotype control antibody on days 27 and 29 after the last immunization. On day 30 after the last immunization, each mouse was challenged with via intravenous inoculation with 10,000 10,000 *Py* WT sporozoites.

### Assessment of effector CD8 T cell function

Liver non-parenchymal cells were isolated from immunized WT C57BL/6 and IFNAR1^−/−^ mice as described above. CD8 T cells were enriched by negative selection using the Easy Sep mouse CD8 T cell enrichment kit (StemCell Technologies, Vancouver BC Canada). For intracellular IFNγ and TNFα staining, 1.5 × 10^5^ liver CD8 T cells were cultured in complete media containing 1× GolgiPlug (BD Biosciences, Franklin lakes NJ) with 5 μg/mL anti-CD3 and 0.5 μg/mL anti-CD28 mAbs to interrogate polyclonal CD8 T cell responses for 6 h. After activation, the cells were washed and stained for cell surface markers and the intracellular markers IFNγ (1:50, XMG1.2, 554413) and TNFα (1:50, MP6-XT22, #506305) using the BD Cytofix/Cytoperm reagent (BD Biosciences, Franklin lakes NJ) according to the manufacturer’s protocol. Samples were acquired on an LSRII flow cytometer (BD Biosciences, Franklin lakes NJ) as described above. For adoptive transfer of CD8 T cells, 2.5 × 10^6^ cells liver CD8 T cells were injected intravenously into naïve C57BL/6 mice. Twenty-four hours after the transfer, each mouse was infected with 1500 *Py* GFPluc sporozoites i.v. and 44 h later, parasite liver burden was quantified by in vivo bioluminescent imaging. To determine whether IFNAR expression intrinsic or extrinsic to CD8 T cells was responsible for the phenotypes observed, liver non-parenchymal cells were isolated from donor naïve OT-1 (C57BL/6-Tg (TcraTcrb)1100Mjb/J) mice as described above. CD8 T cells were enriched by negative selection using the Easy Sep mouse CD8 T cell enrichment kit (StemCell Technologies, Vancouver BC Canada). In all, 100,000 OT-1 cells were then injected into each recipient mouse via the intravenous route. Twenty-four hours later each recipient mouse was immunized with 50,000 *Py fabb/f* ^–^ OVA (LARC GAP OVA). Thirty days after the immunization, livers were harvested from each mouse and liver non-parenchymal cells were isolated for FACS analysis. Samples were acquired on an LSRII flow cytometer (BD Biosciences, Franklin lakes NJ) as described above.

### Liver stage parasite burden by qRT PCR

Livers were harvested from infected C57BL/6 or IFNAR^−/−^ mice into TRIzol reagent (Invitrogen) and homogenized with a Mini-Beadbeater (Biospec, Bartlesville OK). Total RNA was extracted using the Direct-zol RNA miniprep kit (Zymo Research, Irvine CA) and transcribed into cDNA with the QuantiTect Reverse Transcription kit (Qiagen, Venlo Netherlands) according to the manufacturers’ instructions. cDNA samples were amplified using primers for 18S rRNA (forward primer 5′-GGGGATTGGTTTTGACGTTTTTGCG-3′, reverse primer 5′-AAGCATTAAATAAAGCGAATACATCCTTAT-3′) and murine GAPDH (forward primer 5′-CCTCAACTACATGGTTTACAT-3′, reverse primer 5′-GCTCCTGGAAGATGGTGATG-3′). For semi-quantitative PCR a standard curve was generated with reference cDNA. Experimental samples were compared to this standard curve to generate a relative transcript abundance. The qRT PCR was performed on the ABI 7500 Fast Real-time PCR system (Applied Biosystems, Waltham MA).

### Statistical analyses

Data were analyzed using GraphPad Prism Software (Prism 5, La Jolla CA). Kaplan–Meyer patency curves were compared via the log rank Mantel–Cox test. All other statistical significance was determined using a non-paired two-tailed Student’s *t*-test.

### Reporting summary

Further information on research design is available in the Nature Research Reporting Summary linked to this article.

## Supplementary information

Supplementary Information

Reporting Summary

Source Data

## Data Availability

All pertinent data generated or analyzed during this study are included in this published article and its accompanying supplementary information files.
