# Supplementary material for: Innate immunity limits protective adaptive immune responses against pre-erythrocytic malaria parasites
Source: Nat Commun. 2019 Sep 2;10:3950. doi: 10.1038/s41467-019-11819-0 (PMC6718385; doi:10.1038/s41467-019-11819-0)
Supplement: Supplementary file 1 — Supplementary Information [file 41467_2019_11819_MOESM1_ESM.pdf]

Supplementary Figures for Minkah et al 2019, "Innate immunity limits protective adaptive immune responses against pre-erythrocytic malaria parasites”

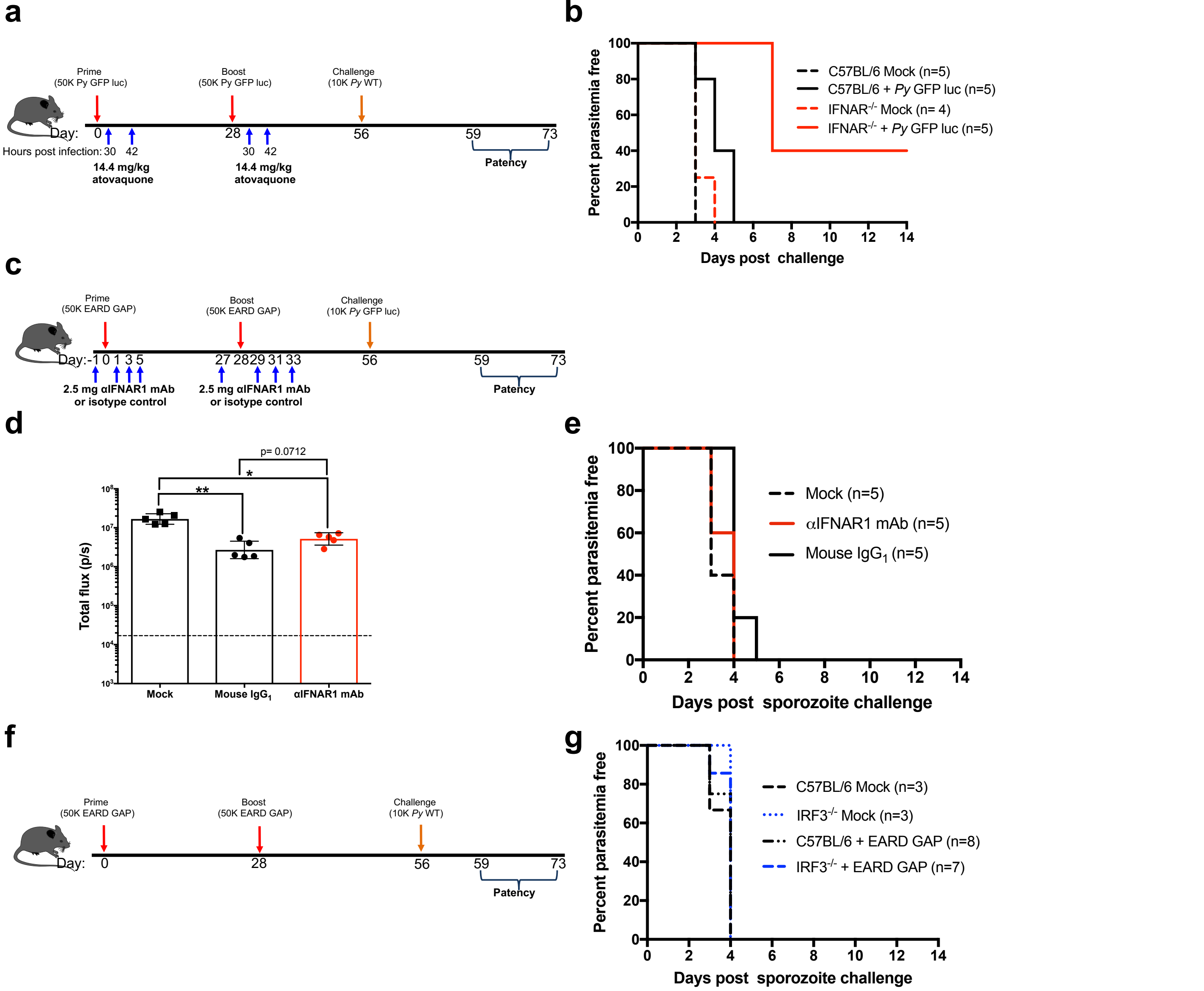

Supplementary Figure 1. Protection is enhanced in IFNAR$^{-/-}$ mice immunized with atovaquone-killed liver stage parasites. (A) Schematic of immunization regimen. **(B)** Examination of blood stage infection in immunized mice after sporozoite challenge 30 days post last immunization**.** Total number of mice in each experiment is shown in the survival curves. **(C)** Schematic of the GAP-immunization regimen. **(D)** Quantification of parasite liver burden 42 hours after challenge with *Py* GFPluc. **(E)** Measurement of blood stage infection in EARD GAP immunized B6 mice treated with isotype control antibody or anti-IFNAR1 antibody. **(F)** Schematic of GAP-immunization regimen. **(G)** Measurement of blood stage infection in EARD GAP immunized B6 mice and IRF3$^{-/-}$ mice. Data from panels B, D, and E represent one of two independent experiments. Data in panel G is compiled from two independent experiments with at least 3 EARD GAP immunized mice per group. Total number of mice in each experiment is shown in the survival curves. Bars represent mean +/- SD. *p<0.05 and **p<0.005 (From unpaired two-tailed Student's t-test) . Components of this figure were created using Servier Medical Art templates, which are licensed under a Creative Commons Attribution 3.0 Unported License; https://smart.servier.com

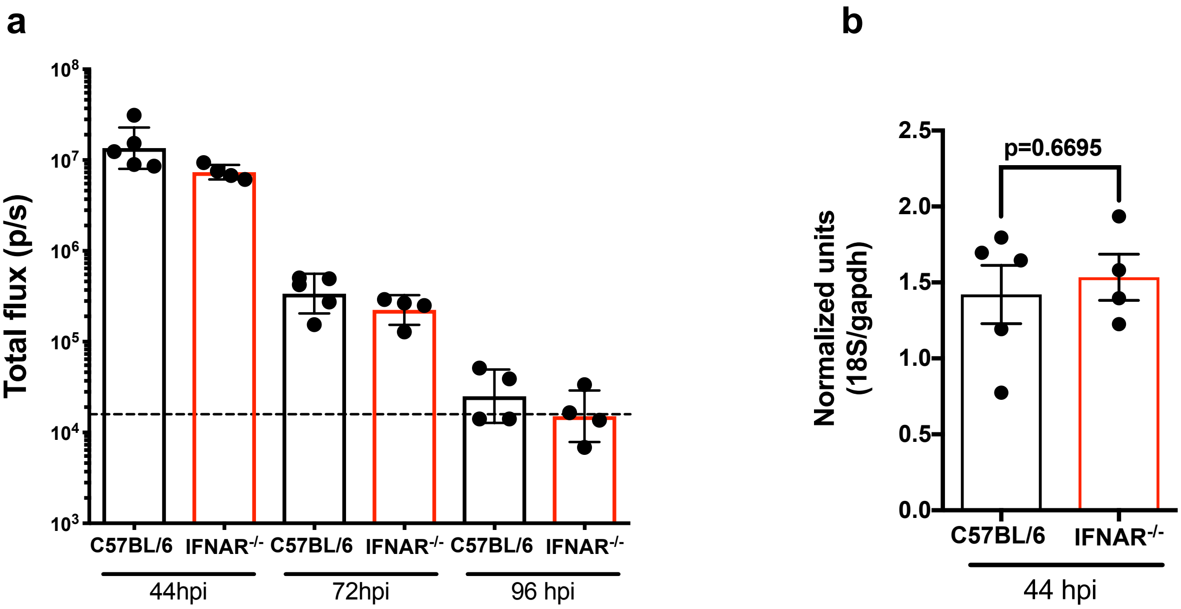

Supplementary Figure 2. Loss of IFN-1 signaling does not impact the magnitude or persistence of *Py* LARC GAP in the liver. B6 mice were infected with 50,00 LARC GAP sporozoites and parasite burden was quantified by *in vivo* bioluminescent imaging **(A)** and qRT-PCR **(B)** Data represent one of two independent experiments with at least 3 mice per group. Each dot represents a single mouse. Bars represent mean +/- SD.

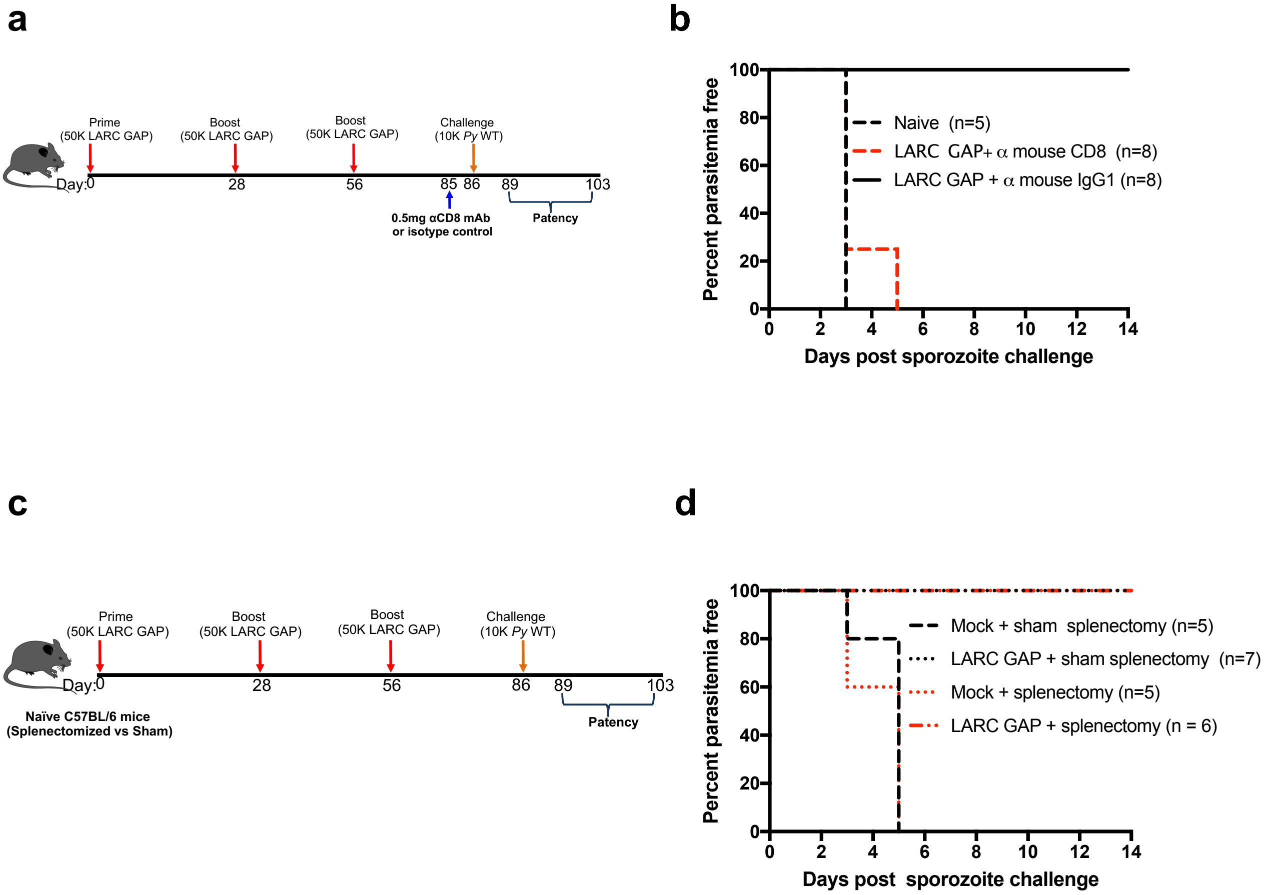

Supplementary Figure 3. Memory CD8 T cells are critical for protection but the spleen is dispensable after LARC GAP immunization (A) Schematic of the GAP-immunization regimen. **(B)** Measurement of blood stage infection in LARC GAP immunized B6 mice treated with isotype control antibody or anti-CD8 antibody. **(C)** Schematic of the GAP-immunization regimen. **(D)** Measurement of blood stage infection in LARC GAP immunized B6 mice. Data from panels B and D are compiled from two independent experiments with at least two mock immunized or naive mice and at least 3 LARC GAP immunized mice in each group. Bars represent mean +/- SD. *p<0.05 and **p<0.005. Total number of mice in each experiment is shown in the survival curves. Components of this figure were created using Servier Medical Art templates, which are licensed under a Creative Commons Attribution 3.0 Unported License; https://smart.servier.com

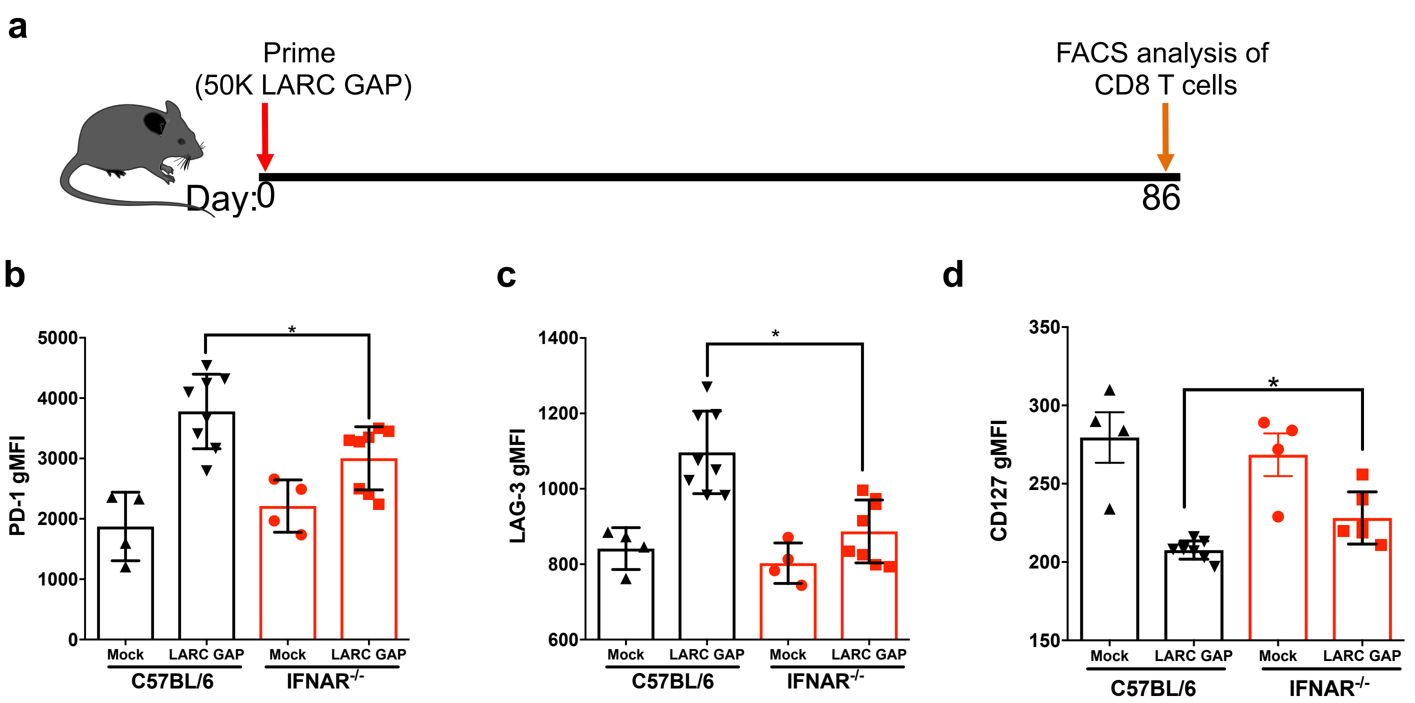

Supplementary Figure 4. The quality of the memory CD8 T cell response is enhanced in immunized IFNAR$^{-/-}$ mice (A) Schematic of the GAP-immunization regimen and experimental setup**(B)** Expression of the co-inhibitory marker, PD-1 on CD44hi CD8 T lymphocytes in immunized IFNAR$^{-/-}$ mice and B6 mice. **(C)** Expression of the co-inhibitory marker LAG-3 on CD44hi CD8 T lymphocytes in immunized IFNAR$^{-/-}$ and B6 mice. **(D)** Expression of the IL-7Rα on CD44hi CD8 T lymphocytes in immunized IFNAR$^{-/-}$ and B6 mice. Data are compiled from two independent experiments with 2 mock-immunized and at least 3 GAP-immunized mice in each group. Each dot represents an individual mouse. Bars represent mean +/- SD. *p<0.05, and **p<0.005 (From unpaired two-tailed Student's t-test) . Components of this figure were created using Servier Medical Art templates, which are licensed under a Creative Commons Attribution 3.0 Unported License; https://smart.servier.com

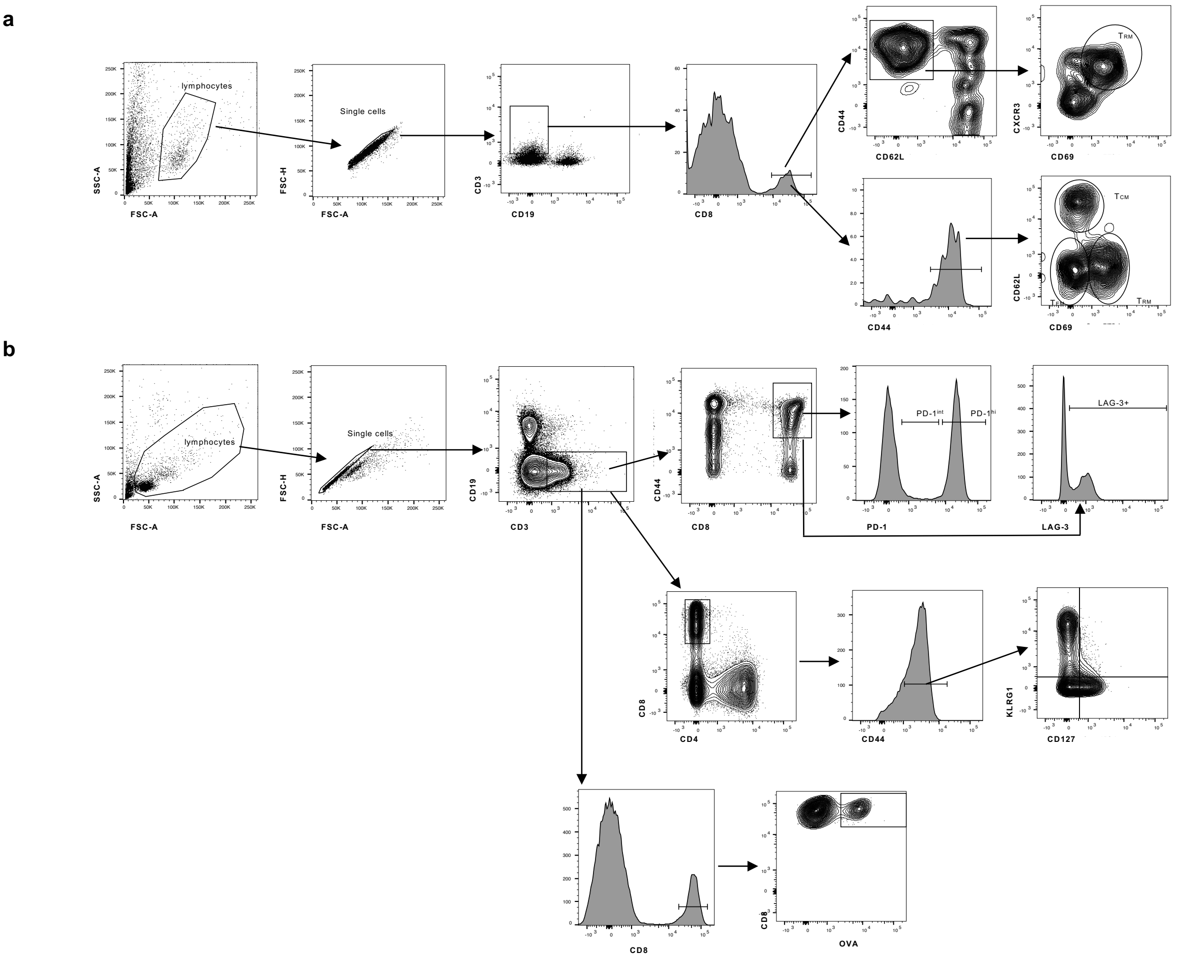

Supplementary Figure 5. Gating strategies used for presented flow cytometric data (A) Gating strategy to identify hepatic memory CD8 T cells presented in figures 2b,2d,2e,3c,3e,3f and 6c **(B)** Gating strategy to identify PD-1, LAG-3 and CD127 expression on memory CD8 T cells, SLEC (KLRG1^{hi} CD127^{lo}) and MPEC (KLRG1^{lo} CD127^{hi}) CD8 T cells and OVA expressing CD8 T cells after immunization with LARC GAP as presented in figures 4,5 and 6 as well as supplementary figures 4b and 4c.
