# Supplementary material for: Innate immunity limits protective adaptive immune responses against pre-erythrocytic malaria parasites
Source: Nat Commun. 2019 Sep 2;10:3950. doi: 10.1038/s41467-019-11819-0 (PMC6718385; doi:10.1038/s41467-019-11819-0)
Supplement: Supplementary file 2 — Reporting Summary [file 41467_2019_11819_MOESM2_ESM.pdf]

nature research

Corresponding author(s): Stefan HI Kappe

Last updated by author(s): Jul 26, 2019

Reporting Summary

Nature Research wishes to improve the reproducibility of the work that we publish. This form provides structure for consistency and transparency in reporting. For further information on Nature Research policies, see Authors & Referees and the Editorial Policy Checklist .

Statistics

For all statistical analyses, confirm that the following items are present in the figure legend, table legend, main text, or Methods section.

n/a	Confirmed	
☐	☒	The exact sample size (n) for each experimental group/condition, given as a discrete number and unit of measurement
☐	☒	A statement on whether measurements were taken from distinct samples or whether the same sample was measured repeatedly
☐	☒	The statistical test(s) used AND whether they are one- or two-sided<br>*Only common tests should be described solely by name; describe more complex techniques in the Methods section.*
☒	☐	A description of all covariates tested
☒	☐	A description of any assumptions or corrections, such as tests of normality and adjustment for multiple comparisons
☒	☐	A full description of the statistical parameters including central tendency (e.g. means) or other basic estimates (e.g. regression coefficient) AND variation (e.g. standard deviation) or associated estimates of uncertainty (e.g. confidence intervals)
☒	☐	For null hypothesis testing, the test statistic (e.g. F, t, r) with confidence intervals, effect sizes, degrees of freedom and P value noted<br>*Give P values as exact values whenever suitable.*
☒	☐	For Bayesian analysis, information on the choice of priors and Markov chain Monte Carlo settings
☒	☐	For hierarchical and complex designs, identification of the appropriate level for tests and full reporting of outcomes
☒	☐	Estimates of effect sizes (e.g. Cohen's d, Pearson's r), indicating how they were calculated

Our web collection on statistics for biologists contains articles on many of the points above.

Software and code

Policy information about availability of computer code

Data collection	none
Data analysis	Flow analysis by Flow Jo software and data analysis and visualization by Graphpad Prism

For manuscripts utilizing custom algorithms or software that are central to the research but not yet described in published literature, software must be made available to editors/reviewers. We strongly encourage code deposition in a community repository (e.g. GitHub). See the Nature Research guidelines for submitting code & software for further information.

Data

Policy information about availability of data

All manuscripts must include a data availability statement. This statement should provide the following information, where applicable:

- Accession codes, unique identifiers, or web links for publicly available datasets
- A list of figures that have associated raw data
- A description of any restrictions on data availability

The source data underlying each figure are provided as a source Data file

Field-specific reporting

Please select the one below that is the best fit for your research. If you are not sure, read the appropriate sections before making your selection.

☒ Life sciences ☐ Behavioural & social sciences ☐ Ecological, evolutionary & environmental sciences

For a reference copy of the document with all sections, see nature.com/documents/nr-reporting-summary-flat.pdf

Life sciences study design

All studies must disclose on these points even when the disclosure is negative.

Sample size	We had previously established in prior publications that at least 4 mice per group is required to obtain statistically robust data. As such this is the minimum number of samples reported used in each experiment
Data exclusions	No data were excluded from these studies.
Replication	Each observation was repeated at least once with the repeated experiments all included
Randomization	For the antibody and drug treatment experiments (CD8 T cell depleteion, CXCR3 depletion,IFNAR blocking antibodies and Atovaquone treatment) WT mice were randomly assigned to the each group. For the WT vs knockout mouse comparisons, mice at similar ages and of the same sex were randomly chosen for each experiment
Blinding	The geneotypes of mice used were known prior to immunization thus there was no blinding in this study

Reporting for specific materials, systems and methods

We require information from authors about some types of materials, experimental systems and methods used in many studies. Here, indicate whether each material, system or method listed is relevant to your study. If you are not sure if a list item applies to your research, read the appropriate section before selecting a response.

Materials & experimental systems

n/a	Involved in the study	
☐	☒	Antibodies
☒	☐	Eukaryotic cell lines
☒	☐	Palaeontology
☒	☐	Animals and other organisms
☒	☐	Human research participants
☒	☐	Clinical data

Methods

n/a	Involved in the study	
☒	☐	ChIP-seq
☐	☒	Flow cytometry
☒	☐	MRI-based neuroimaging

Antibodies

Antibodies used	Isolated cells were labeled with mAb specific for CD3 (clone 145-2C11), CD4 (clone GK1.5), CD8 (clone 536.7), CD19 (clone 6D5), CD44 (clone IM7), , CD62L (clone MEL-14), CD69 (clone HI.2F3), PD-1/CD279 (clone 29F.1A12), CD223/LAG-3 (clone C9B7W), CD127/IL-7R (clone A7R34), KLRG1(clone 2F1), and CXCR3 (clone CXCR3-173) all from biolegend. OVA-specific CD8 T cells were identified using an MHC-I tetramer against the H-2kb peptide SIINFEKL (NIH tetramer core).
Validation	These antibodies have been generated and extensively validated against murine targets

Flow Cytometry

Plots

Confirm that:

☒ The axis labels state the marker and fluorochrome used (e.g. CD4-FITC).

☒ The axis scales are clearly visible. Include numbers along axes only for bottom left plot of group (a 'group' is an analysis of identical markers).

☒ All plots are contour plots with outliers or pseudocolor plots.

☒ A numerical value for number of cells or percentage (with statistics) is provided.

Methodology

Sample preparation	For the analysis of liver memory CD8 T cells, livers of immunized mice harvested four weeks after the last immunization were cut into small pieces, mechanically disrupted using the plunger of a 10 mL syringe and strained through a 100 µm nylon filter. The nonparenchymal cell fraction was isolated on iodixanol gradient.For the phenotypic analysis of splenic lymphocytes, mouse spleens were harvested and homogenized to form single cells suspensions of splenocytes. Red blood cells were removed by lysis with ACK lysis buffer and the splenocytes strained through a 100µm nylon filter. For the analysis of peripheral memory CD8 T cells, heparinized blood was collected from immunized mice and treated with ACK lysis buffer to remove red blood cells. Isolated cells were labeled with monoclonal antibodies specific for markers of interest

Instrument	Labeled cells were run on an LSRII flow cytometer (BD Biosciences, Franklin lakes NJ)
Software	Data was acquired using FACS DIVA and analysed using FlowJo software
Cell population abundance	No cells were sorted for these studies
Gating strategy	The gating strategy for each population is described in the text. Briefly samples were gated on lymphocytes by FSC and SSC followed by a discrimination between CD3+ T cells and CD19 positive B cells. CD8 T cells were then gated out of the CD3+ population. Subsequent populations were then gated out of the CD8 T cell population

☒ Tick this box to confirm that a figure exemplifying the gating strategy is provided in the Supplementary Information.
